# Lavender hydrosol analysis using UV spectroscopy data and partial least squares regression

**DOI:** 10.1016/j.mex.2025.103304

**Published:** 2025-04-05

**Authors:** Sára Preiner, Bálint Levente Tarcsay, Dóra Pethő, Norbert Miskolczi

**Affiliations:** Research Centre for Biochemical, Environmental and Chemical Engineering, University of Pannonia, Egyetem st.10, Veszprém, 8200, Hungary

**Keywords:** Hydrosol analysis, Lavender, UV–Vis, Partial least squares regression, *Lavender hydrosol analysis using UV–Vis spectroscopy and partial least squares regression*

## Abstract

The aim of our work was to estimate the composition of hydrosol produced as a byproduct of lavender steam distillation using UV–Vis spectrophotometry in the 200–600 nm wavelength range through a machine learning algorithm. The dissolved components of lavender essential oil (EO) from lavender hydrosol samples were extracted via liquid-liquid extraction, using three different solvents (pentane, heptane and diethyl ether). The UV–Vis absorbance spectra of the extracts were recorded and the composition analyzed using GC–MS. The composition data obtained allowed for the calculation of changes within the quantities of different EO components in the samples.

The partial least squares regression technique (PLS) was utilized to establish a connection between changes in the composition of the hydrosol and the changes in the UV–Vis spectra. After optimization the established PLS model showed an $R^{2}$ score above 0.95 for the prediction of hydrosol composition changes during cross-validation. The model can thus be utilized as a soft sensor to infer extracted mass of EO components and characterize the composition of hydrosol during the process directly from UV–Vis spectra.

• Investigation of lavender water and extract using UV–Vis spectrophotometry
• GC–MS analysis of extracts
• PLS model development for composition estimation based on spectra

## Graphical abstract

**Figure:**
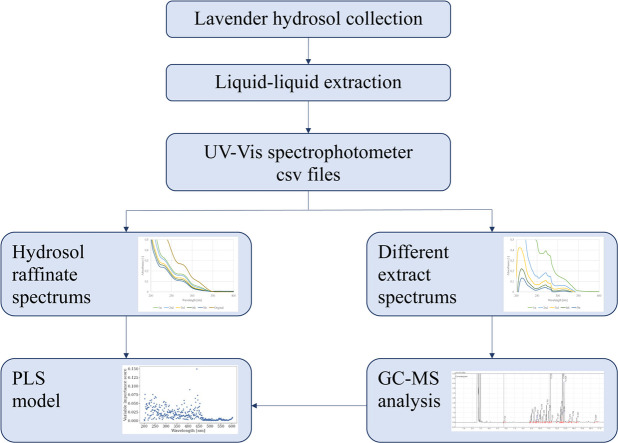

Specifications table

**Table:** 

Subject area:	Chemical Engineering
More specific subject area:	*Composition detection using soft sensors*
Name of your method:	*Lavender hydrosol analysis using UV–Vis spectroscopy and partial least squares regression*
Name and reference of original method:	*–*
Resource availability:	*–*

## Background

In this work a method is developed to infer composition changes within lavender hydrosol based on UV–Vis spectra and partial least squares (PLS) regression. Essential oil (EO) isolated from lavender is one of the most used aromatic products which can be found in food, pharmaceuticals and cosmetic industries just like in aromatherapy due to its pleasant fragrance and calming effect on the nervous system [1,2].

Its extensive usage can be attributed to the chemical composition of the EO. These oils can contain more than one hundred components [3]. Linalool and linalyl acetate are the two main components of lavender EO but eucalyptol, α-terpineol, terpinen-4-ol, lavandulol and lavandulyl acetate are also important compounds. The amount of these components depends on the lavender type and extraction technique [4,5].

In industrial applications steam distillation is the most commonly used method to extract EO [6,7]. Through steam distillation of lavender, huge amounts of aqueous solution remain as industrial byproducts called hydrosol or floral water. Several studies investigated the composition and effect of the hydrosols which can widely be used in different sectors of the pharmaceutical, food and cosmetics industry [8]. Therefore, it is required to develop methods to effectively estimate the composition of hydrosols with a minimal number of experimental procedures to minimize the costs of analysis. In this work the authors developed a method to calculate the changes in EO component mass of lavender hydrosol using UV–Vis analysis and partial least squares regression (PLS).

The investigation of the hydrosols in terms of composition and different properties were implemented by liquid-liquid extraction and gas chromatography analysis of the extract [9,10], while biological properties, application possibilities, aromatic, antioxidant and antimicrobial effects of lavender aromatic water were determined [11,12].

The aim of our work was to develop a faster and cheaper method to quantify the main components of lavender hydrosol using soft sensors. UV–Vis spectrophotometry was used to define the changes in the main components of the floral water. The volatile components of the hydrosol were separated by liquid -liquid extraction. The original hydrosols and the extracts were analyzed with UV–Vis spectrophotometry. To specify the main components the extracts were analyzed by GC–MS and compared to the spectra of the samples.

Subsequently the partial least squares regression (PLS) was used to establish a link between changes in the composition of the hydrosol (based on GC–MS) and the changes in the UV–Vis spectra [13]. Using the PLS regression as a soft sensor the GC–MS step to determine the composition changes within the hydrosol could be eliminated and the composition changes could be directly inferred from changes in the UV–Vis spectra of hydrosol.

The high accuracy PLS model developed ($R^{2}>0.95$ during five-fold cross-validation) enabled the elimination of the use of GC–MS and thus simplified the composition evaluation process [14].

## Method details

### Sample collection and analysis

Various types of lavender hydrosols were investigated to establish the composition estimation method. Industrial samples were collected in the city of Tihany (46.90891°N 17.87923°E), which belongs to the main lavender essential oil producing region of Hungary. Lavender plants were collected from different production areas there to perform laboratory scale experiments. The plant samples were dried until constant mass and were chopped before the steam distillation measurements to uniform size distribution. The measured sample (170 g) and the steam flowrate was the same in case of every experiment. After the steam distillation methods, the lavender hydrosol samples were collected and analyzed by UV–Vis spectroscopy and liquid-liquid extraction with different extractants (pentane, diethyl ether, heptane). All in all, 22 (S1-S22) samples were produced and evaluated.

**Fig. 1:**
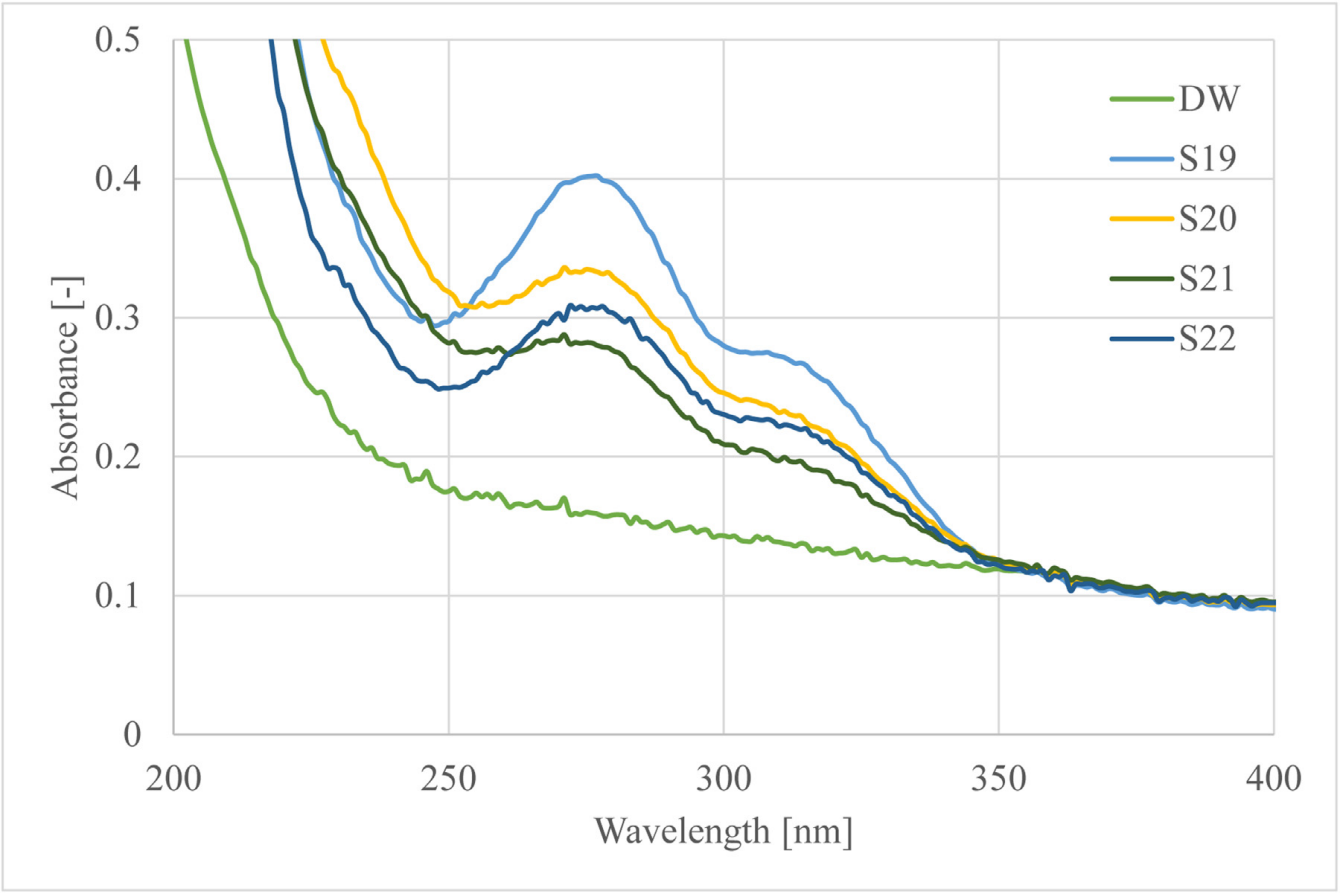
UV–Vis spectrum of distilled water (DW) and lavender hydrosols of samples S19-S22.

Our goal was to define the main EO components and their concentrations within the hydrosol. The original samples were tenfold diluted. 0.1 ml of the sample was measured using an automatic pipette which was transferred to a 10 cm^3^ standard flask and filled to mark. The cuvette was filled with the examined samples and their spectra was taken with Thermo Scientific Genesys 150 UV–Vis spectrophotometer. The blank solutions were distilled water for the hydrosol samples and pentane, diethyl ether and heptane for the extracts. The spectra were taken between 200 and 600 nm range wavelengths. The main differences were seen in the UV domain between 200 and 400 nm. The spectrum of different hydrosols and distilled water (DW) can be seen on Fig. 1.

The spectra were corrected with the absorbance of distilled water. The absorbances were read at the maximum change of the spectra which were at wavelength 278 nm. Changes can be seen in the spectrum at the given wavelength so it can be inferred that every sample contains different amounts of the EO components.

To find out which are the main components of the hydrosols, a given volume of the hydrosol (50 cm^3^) was extracted with different solvents. Three different solvents, heptane, pentane and diethyl‑ether were used as extractants for the measurements. The liquid-liquid extraction was performed in a separation funnel, the contact between the two phases lasted 10 min every time. In every case 50 cm^3^ of the hydrosol sample was extracted with 10 cm^3^ solvent. The extractions were carried out in five stages for the heptane solvent and in one stage for pentane and diethyl‑ether as for the latter two spectra did not show significant changes after the first extraction step. For the heptane extraction fresh solvent was used in every stage. The extracts were also diluted tenfold or more if the extracted amount was too high and the spectrum was not relevant.

Measurement samples S1-S5 and S6-S10 were performed with industrial hydrosol from the year 2024 and 2023 respectively. Each sample composition was evaluated during one of the stages of five-stage heptane extraction method. The same industrial hydrosols were used for S11-S12 and S17-S18, while the hydrosols from laboratory experiments (with lavender plants from different producing areas) were used for S13-S16 and S19-S22. These latter samples were extracted with pentane and diethyl‑ether respectively.

**Fig. 2:**
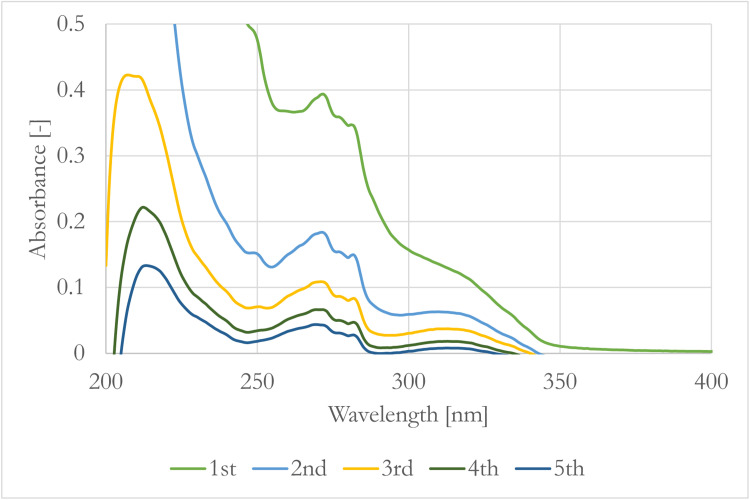
Spectra of the heptane extracts in every stage.

**Fig. 3:**
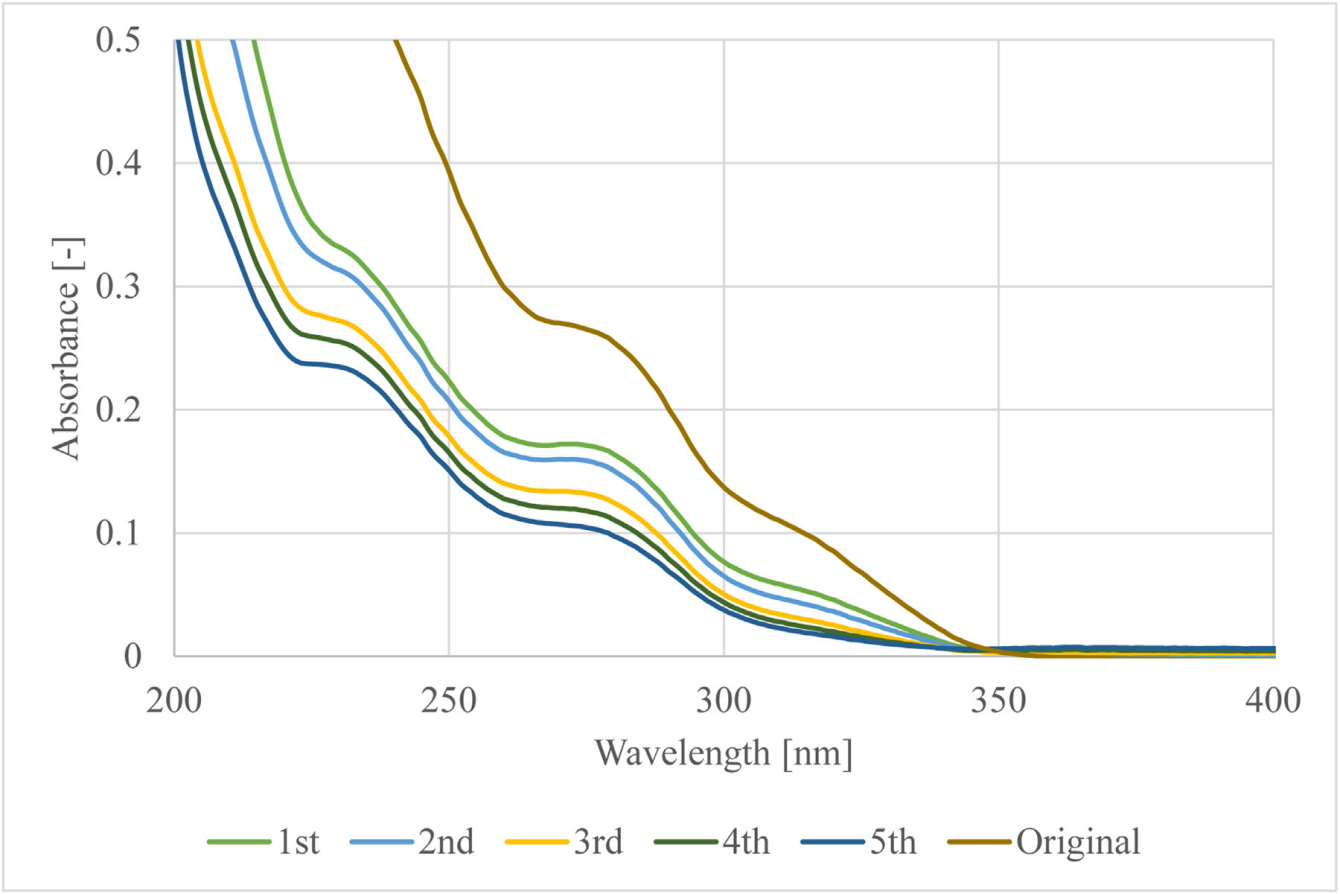
Spectra of the original hydrosol and the raffinates in every stage.

To monitor the extractable components, the extracts and the raffinates were analyzed with UV–Vis as well. The spectra of the extracts (S1-S5) in five stages can be seen on Fig. 2 while the raffinate spectra are shown in Fig. 3.

Fig. 2 shows how the amount of extracted components changes in the solvent through the stages. After the first extraction the maximum absorbance value of the spectrum was around 0.4 while after the second extraction it did not reach the 0.2 absorbance and it continuously decreased in every stage. The same information can be read about Fig. 3. The absorbance difference of the original and once extracted hydrosol is around 0.1 at 277 nm, while there are no such big differences between the last four extracted hydrosols.

The extracts were collected in every stage and their composition was analyzed by GC–MS.

Agilent model 5975C gas chromatograph was used to perform the analysis. The GC separation was achieved on an HP5 type column (30.0 *m* × 0.25 mm; film thickness 0.25 µm). The injector and detector temperatures were set to 250 °C. 0.1 µL of the sample was injected on the column. The column was programmed at 10 °C/min heating rate from 50 °C to 250 °C. The samples were not diluted through the examination and in this way 9 main components were identified. These are α-pinene, β-trans-ocimene, β-cis-ocimene, linalool, octene-1-ol, camphor, terpinen-4-ol, hexyl butyrate and lavandulyl acetate. The weight percentage composition (m/m %) of the extracted oil in case of different extractants can be seen in Table 1, which shows that linalool, terpinene-4-ol and hexyl butyrate are the main components.

**Table 1:** Composition of the extracted oil from the same hydrosol in case of different extractants (S6, S12, S18, first stage).

Component	Composition (m/m %)		
	Pentane	Diethyl ether	Heptane
α-pinene	2.28	1.84	0.4
β-trans-ocimene	6.33	5.49	4.7
β-cis-ocimene	1.52	1.17	0
linalool	43.80	41.65	39.66
octen-1-ol	2.64	2.79	2.65
camphor	3.75	3.71	4.28
terpinen-4-ol	16.14	15.04	18.36
hexyl butyrate	6.70	6.70	11.48
lavandulyl acetate	0.37	0.53	0.33

Table 1 shows the extracted oil composition but to calculate the exact amount of these components in the hydrosols, the amount of the solvent in the extract should not be ignored. The information in Table 2 was used to calculate the amount of different types of components in the extract.

**Table 2:** Composition of the extract from the same hydrosol in case of different extractants (S6, S12, S18, first stage).

Component	Composition (m/m %)		
	Pentane	Diethyl ether	Heptane
solvent	99.283	97.404	99.490
α-pinene	0.008	0.033	0.001
β-trans-ocimene	0.010	0.040	0.024
β-cis-ocimene	0.006	0.026	0.001
linalool	0.299	0.970	0.212
octen-1-ol	0.007	0.025	0.014
camphor	0.015	0.063	0.020
terpinen-4-ol	0.152	0.491	0.102
hexyl butyrate	0.032	0.116	0.056
lavandulyl acetate	0.005	0.025	0.002
other	0.183	0.807	0.078

After establishing the composition changes during each step of the liquid-liquid extraction process partial least squares (PLS) regression was used to establish a connection between changes in the UV–Vis spectra, the type of employed solvent and the changes in the mass of lavender EO components within the hydrosol. For a given solvent i the change of mass (Δm) within the water for a specific EO component j was calculated using Eq. (1).

(1) $$\Delta m_{i,j}=x_{i}\rho_{j}V_{j}$$

In Eq. (1). $x_{i}$ refers to the mass fraction of the *i* th component calculated as per the results of Table 2. The variable $\rho_{j}$ is the density of the extractant used for the specific sample and $V_{j}$ is the volume of the employed extractant.

The changes within the mass of the EO components within the hydrosol were calculated for each sample and different extractants. The results are shown in Table 3, where DEE refers to the solvent diethyl ether.

**Table 3:** Changes within the mass of EO components in hydrosol during the use of different solvents.

Solvent	Sample number	α-pinene (g)	β-trans-ocimene (g)	β-cis-ocimene (g)	linalool (g)	octen-1-ol (g)	camphor (g)	terpinen-4-ol (g)	hexyl butyrate (g)	lavandulyl acetate (g)
Heptane	S1	0.078	0.084	0.089	0.092	0.093	0.093	0.090	0.086	0.080
Heptane	S2	0.008	0.009	0.009	0.010	0.011	0.012	0.013	0.014	0.016
Heptane	S3	0.007	0.007	0.007	0.008	0.008	0.009	0.009	0.010	0.011
Heptane	S4	0.002	0.002	0.002	0.002	0.003	0.003	0.003	0.003	0.003
Heptane	S5	0.003	0.003	0.003	0.003	0.003	0.003	0.003	0.004	0.004
Heptane	S6	0.070	0.076	0.082	0.086	0.089	0.089	0.087	0.083	0.079
Heptane	S7	0.015	0.016	0.017	0.019	0.020	0.022	0.024	0.025	0.027
Heptane	S8	0.004	0.004	0.004	0.004	0.005	0.005	0.005	0.006	0.006
Heptane	S9	0.005	0.005	0.005	0.005	0.006	0.006	0.006	0.007	0.007
Heptane	S10	0.002	0.002	0.002	0.002	0.002	0.002	0.002	0.002	0.003
Pentane	S11	0.040	0.042	0.044	0.046	0.048	0.049	0.050	0.052	0.053
Pentane	S12	0.019	0.021	0.022	0.020	0.020	0.020	0.018	0.017	0.015
Pentane	S13	0.014	0.015	0.017	0.016	0.017	0.016	0.016	0.016	0.015
Pentane	S14	0.039	0.042	0.044	0.046	0.048	0.049	0.050	0.052	0.053
Pentane	S15	0.023	0.026	0.028	0.030	0.032	0.035	0.036	0.038	0.039
Pentane	S16	0.009	0.011	0.013	0.011	0.012	0.013	0.013	0.012	0.011
DEE	S17	0.026	0.029	0.031	0.033	0.036	0.039	0.041	0.043	0.045
DEE	S18	0.024	0.027	0.030	0.032	0.035	0.038	0.040	0.042	0.044
DEE	S19	0.037	0.040	0.043	0.044	0.047	0.050	0.051	0.053	0.053
DEE	S20	0.045	0.047	0.051	0.052	0.055	0.057	0.059	0.061	0.062
DEE	S21	0.035	0.038	0.041	0.043	0.046	0.049	0.051	0.053	0.055
DEE	S22	0.025	0.027	0.029	0.028	0.030	0.031	0.031	0.031	0.031

Using the 22 obtained samples the PLS regression model was established. The type of solvent and the changes in the UV–Vis spectra were used as input variables while the changes in EO component mass were used as predicted variables.

**Fig. 4:**
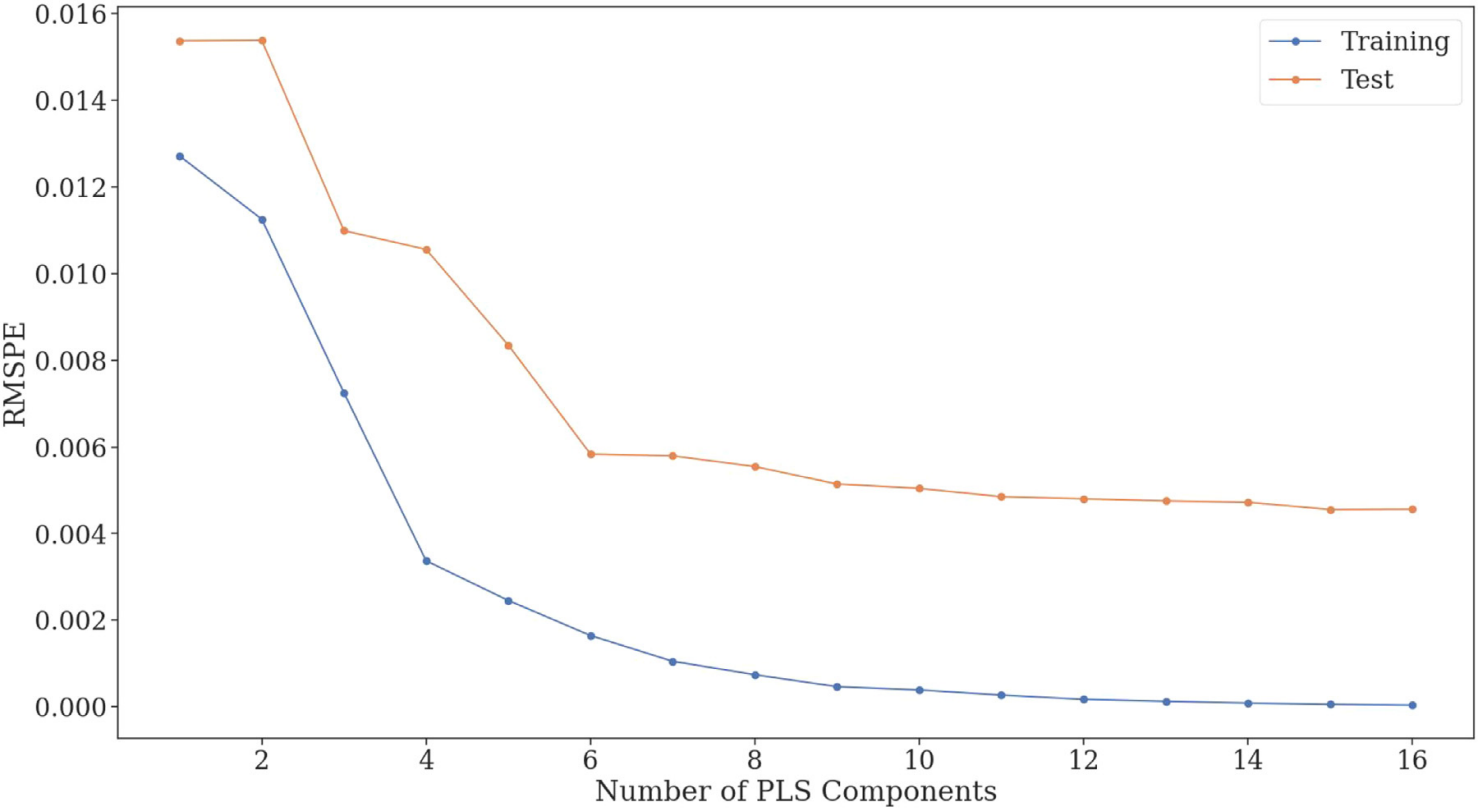
Average RMSPE [-] of the PLS model to training and test data as a function of PLS component number during five-fold cross-validation.

During the dimensionality reduction step the optimal number of latent variables

(PLS components) was evaluated using five-fold cross-validation. The $R^{2}$ and RMSPE (root mean square percentage error) metrics were utilized to evaluate the fit of the model to the data. The model fit as a function of PLS component number in terms of RMSPE can be seen in Fig. 4. The figure shows that after a certain point adding more latent variables to the PLS model its fit to the test data does not increase significantly. Therefore, an optimal PLS component number of 15 was chosen during further investigation, at this point the root mean square percentage error of the fit was 0.004 or 0.4 %.

**Fig. 5:**
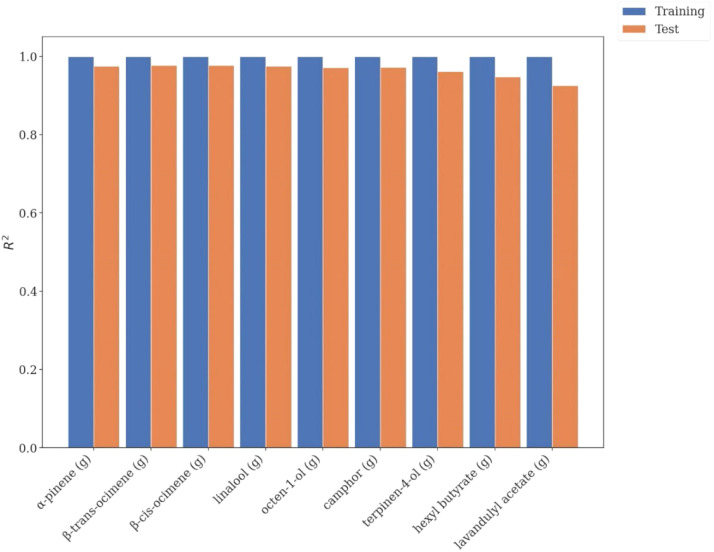
Average $R^{2}$ scores during the five-fold cross-validation for each individual EO component.

In case of each predicted EO component the cross-validated $R^{2}$scores are displayed individually on Fig. 5. The results showcase that for each EO component the model was able to accurately predict the change of component mass within the hydrosol based on the change in the spectra.

**Fig. 6:**
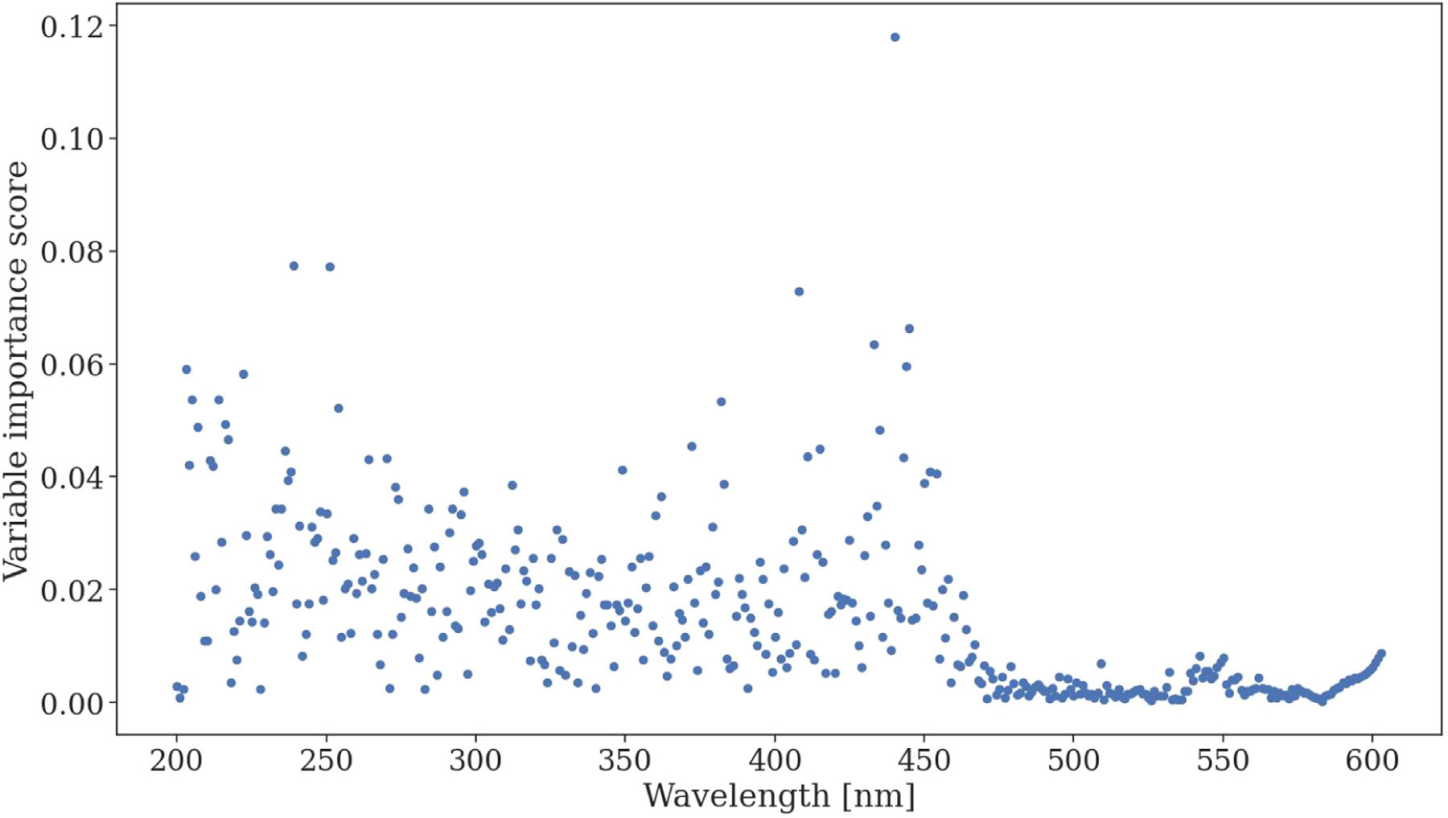
Variable importance of different UV–Vis wavelengths for determining changes in hydrosol EO component masses.

The variable importance among the predictor variables was observed for each utilized wavelength and scored. The variable importance scores are displayed in Fig. 6. The results indicate that wavelengths between 200 and 450 nm are most significant for determining the composition changes, higher wavelength spectra may be omitted during future use of the model.

## Method validation

**Fig. 7:**
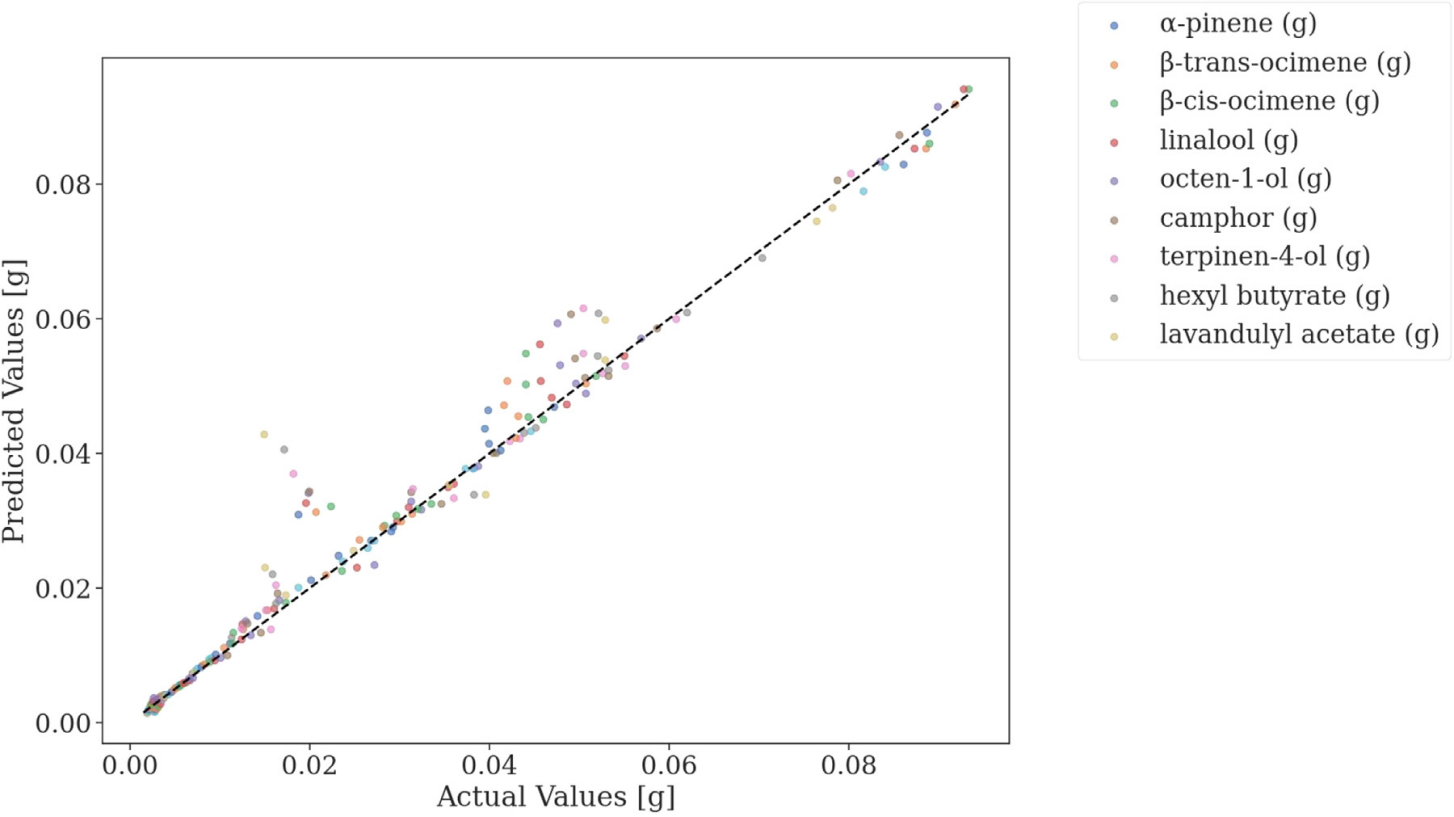
*Fit* of the optimized PLS model to the test data during five-fold cross-validation.

Using the optimized PLS model the mass change in the EO components within the water was predicted for each solvent during the extraction process based on the change in the UV–Vis spectra. The predicted and measured mass changes in the EO components during the five-fold cross-validation process using the optimized model are shown in Fig. 7.

As the $R^{2}$ score indicated the model has high accuracy in predicting the changes in individual EO components within the hydrosol during the extraction process for the different solvents. Greater deviations can only be observed in the case of lavandulyl acetate and hexyl butyrate being 0.02 g in magnitude.

**Fig. 8:**
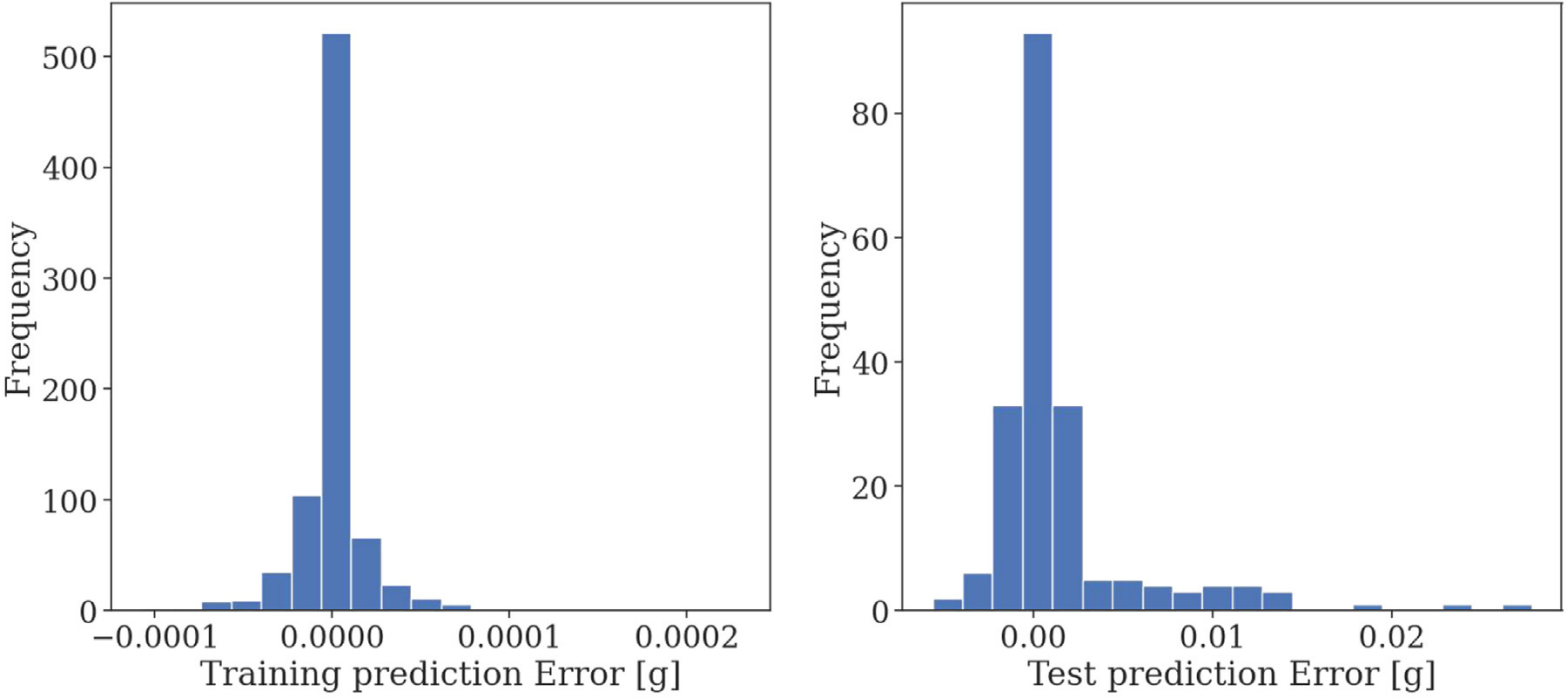
Distribution of the PLS model errors during the five-fold cross-validation process.

To evaluate the appropriate nature of the PLS model for the data the distribution of prediction errors for all component mass changes during the cross-validation process were evaluated as well. These errors show an approximately normal distribution indicating that the model error is random in nature and not due to neglected terms during modeling. The distribution of the error terms can be seen in Fig. 8.

The results indicate that the PLS model is adequate in replacing the GC–MS step for estimating the composition changes within lavender hydrosol during fluid extraction. The model may be used for industrial purposes to evaluate the composition of lavender hydrosol derived from steam as well as hydrodistillation using UV–Vis spectroscopy without a need for more in-depth analytical techniques for composition determination.

## Limitations

The model applies to the solvents used during the measurements. In case of different solvent usage, additional data must be provided to the model.

## Ethics statements

This work did not involve human subjects, animal experiments or data from social networks

## CRediT author statement

**Sára Preiner:** Conceptualization, Methodology, Investigation, Writing - Original draft preparation, **Bálint Levente Tarcsay:** Conceptualization, Data curation, Visualization, Writing - Original draft preparation, **Dóra Pethő:** Supervision, Writing- Reviewing and Editing, **Norbert Miskolczi:** Supervision, Writing - Reviewing and Editing,

## Declaration of competing interest

The authors declare that they have no known competing financial interests or personal relationships that could have appeared to influence the work reported in this paper.

## Data Availability

Data will be made available on request.
